# Comparison of Two Surgical Techniques for the Treatment of Equine Hindlimb Proximal Suspensory Desmopathy

**DOI:** 10.3390/ani15172598

**Published:** 2025-09-04

**Authors:** Kendra D. Freeman, M. Norris Adams, Allison E. Salinger, Nathaniel A. White, Jennifer G. Barrett

**Affiliations:** Marion duPont Scott Equine Medical Center, Virginia-Maryland College of Veterinary Medicine, Virginia Tech, 17690 Old Waterford Rd., Leesburg, VA 20177, USA

**Keywords:** suspensory ligament, desmopathy, desmitis, neurectomy, desmoplasty, fasciotomy, lameness

## Abstract

The objective of this retrospective study was to compare outcomes after desmoplasty with fasciotomy (DF) against deep branch of the lateral plantar neurectomy with fasciotomy (NF) for lameness due to proximal suspensory desmopathy in the hindlimb. Medical records from 141 horses with proximal suspensory desmopathy treated by either desmoplasty with fasciotomy or deep branch of the lateral plantar neurectomy with fasciotomy were reviewed. Follow-up after surgery to determine resolution of the lameness and return to use was obtained from medical records, as well as referring veterinarians, owners and trainers by telephone conversations and/or email. Long-term outcomes were obtained for 109 horses. There was no significant difference in resolution of lameness between the two techniques (79% DF vs. 88% NF), or in the percentage of horses returning to work 1–3 years post-operatively (83% DF vs. 93% NF). Horses treated with DF had higher ultrasonography scores compared with horses treated with neurectomy/fasciotomy. Horses treated with DF took longer to return to work compared to NF and received more non-surgical treatments in the recovery period. Post-operative complications were minimal with either technique. Use of either surgical technique for hindlimb proximal suspensory desmopathy can result resolution of lameness and return to athletic use.

## Simple Summary

This study compared two different surgical treatments of a common problem causing lameness in horses: hind limb suspensory ligament injury in 141 horses. One surgical technique is minimally invasive, and splits the ligament and the overlying tissue that constricts the ligament desmoplasty with fasciotomy (DF). The other surgery cuts the overlying constrictive tissue, but also cuts the nerve that specifically goes into the suspensory ligament deep branch of the lateral plantar neurectomy with fasciotomy (NF). Both techniques resulted in soundness at similar levels, and in return to work at similar levels. One technique (DF) was used more often in horses with evidence of tearing of the suspensory ligament, which may indicate surgeon preference. Neither surgery was associated with serious operative complications; however, three horses had their suspensory ligaments loosen too much resulting in dropped fetlocks.

## 1. Introduction

Suspensory desmopathy is a frequently documented cause of lameness in performance horses; proximal suspensory desmopathy is the most frequent site of injury in dressage horses [1,2]. Desmopathy of the proximal suspensory ligament in the hindlimb has a poorer prognosis for resolution compared to suspensory desmopathy in the forelimb [3]. The hindlimb proximal suspensory ligament originates as a broad attachment to the proximal plantar aspect of the third metatarsal bone with smaller attachments originating from the distal tarsal bones [1]. The proximal portion of the ligament is bound by the second and fourth metatarsal bones as well as a dense plantar fascia, preventing direct palpation of the non-diseased ligament [4]. The ligament is composed predominately of collagenous tissue, with 2–11% of the tissue composed of muscle [5]. The hindlimb proximal suspensory ligament is innervated by the deep branch of the lateral plantar nerve, which enters the ligament approximately 17 mm distal to the palpable head of the fourth metatarsal bone [6]. Occasionally a second branch is present at this location [6].

Hindlimb proximal suspensory desmopathy is not a facile clinical diagnosis. It can either present with an acute onset of obvious lameness or as an insidious onset of lameness. Because many horses are affected bilaterally, the presenting complaint may be poor performance rather than an obvious lameness [7]. Horses can be positive to both full hindlimb flexion and upper hindlimb flexion [8] and the lameness can be identified under varying conditions; straight line on a firm surface [9], in a circle [1] and under saddle [3]. Several techniques have been described to desensitize the proximal suspensory ligament including the high six-point block [10], the subtarsal block [10] and direct infusion in the location of the deep branch of the lateral plantar nerve (DBLPN) [6,11]. Blocking sensation of this nerve has been shown to be less likely to inadvertently infiltrate nearby synovial structures [6]; however, in a study by Labens et al. this technique failed to localize pain to the proximal suspensory ligament in six of 38 cases [11]. Diffusion of local anesthetic is possible, further complicating the diagnosis [12].

In cases of proximal suspensory desmopathy, ultrasonographic findings may include increased size as determined by dorsoplantar thickness or cross-sectional area, focal or diffuse decreased echogenicity, changes in fiber pattern, poor demarcation of the margins of the ligament and/or focal mineralization [1,3,13]. Intra- and inter-operator variability, and poor correlation with magnetic resonance imaging are limitations of diagnostic ultrasonography [11,14]. Nuclear scintigraphy and radiography are of limited diagnostic value because bone involvement at the origin is not always present [3,7,9,15]. Magnetic resonance imaging is the gold standard for identifying and confirming the presence of suspensory ligament injury and inflammation; however, it is not always available, practical, and affordable [13,16]. PET imaging is a newer modality that may prove useful in diagnosis, especially in combination with other imaging modalities [13].

Non-surgical management of proximal suspensory desmopathy may include a period of rest followed by gradual return to work, extracorpeal shock wave therapy [17] (ECSWT), regenerative therapies [18,19,20], and injection of bioscaffold material [17]. A nine-week program of rest and gradual increase in exercise was successful in returning 40% of horses to work, with only 12.5% of horses able to remain in work without recurrent lameness [9]. A single study using unprocessed bone marrow aspirate injections returned 84% of cases to work, although some of these horses were concurrently treated with a fasciotomy procedure and both front and hind suspensory ligaments were included in the study [18].

Surgical treatments of hindlimb proximal suspensory desmopathy include desmoplasty and fasciotomy [21] (DF) with a 15 week rehabilitation protocol, and deep branch of the lateral plantar neurectomy and DBPLN neurectomy combined with fasciotomy (NF) with a 60 day rehabilitation protocol. The goal of the combined desmoplasty and fasciotomy procedure is to decompress acute or static hypoechoic lesions and promote new blood supply to chronic lesions [21]. Of the 23 cases for which follow up of DF was available, 87% (20 horses) returned to work. Neurectomy was initially reported by Toth et al. with 62.5% of horses returning to intended use [22]. The success rate of the neurectomy procedure was improved with the addition of the fasciotomy with 77.8% of horses returning to work for at least one year [15]. Following this treatment some horses remained lame, with lameness isolated to the proximal suspensory ligament. Additionally, iatrogenic damage to the suspensory ligament during the fasciotomy procedure has been documented, and a specialized fasciotome has reduced the incidence of this occurrence [15,23].

These two surgical procedures are commonly performed in our hospital, and the purpose of this study was to compare the outcomes of these two surgical techniques with their specific rehabilitation protocols in a retrospective study. We hypothesized that the number of horses that returned to work following surgical treatment would not be different between groups.

## 2. Materials and Methods

### 2.1. Case Selection

This study was a retrospective analysis of veterinary clinical cases, and best practice veterinary care was followed. As such, this study is exempt from requiring ethical approval. Written informed consent was provided by the owners of the animals for the analysis. Medical records of all horses admitted to the Marion duPont Scott Equine Medical Center from 2007 to 2013 with the diagnosis of hindlimb proximal suspensory desmopathy were reviewed. Horses were included in the study if the diagnosis of hindlimb proximal suspensory desmopathy was made based on the findings of lameness examination, local anesthesia and/or imaging via ultrasonography and the treatment consisted of either the DF or NF. Horses that did not block to the suspensory via nerve block or local infiltration were excluded from the study. One horse that had both surgeries during the same anesthetic event was excluded. If a horse had multiple surgeries under separate anesthetic events, only the first surgery was included in the DF versus NF comparative analysis, and those were counted as not sound/not in work for outcome analysis.

### 2.2. Medical Record Review

Information recorded from the medical record included signalment, history, duration of lameness, results of the lameness examinations, results of diagnostic imaging studies (ultrasonography, nuclear scintigraphy, magnetic resonance imaging), previous treatments, which surgical procedure was performed and additional treatment of the ligaments with intralesional injection of platelet rich plasma, stem cells, and/or extracorporeal shockwave therapy. Horse athletic use was recorded when available.

### 2.3. Ultrasonography Findings

Medical records had findings that corresponded with 8 categories, that graded by the following scale: 0 = Within Normal Limits, 1 = Mottled fiber pattern, 2 = Enlarged without changes in fibers, 3 = Enlarged and Mottled, 4 = Hypoechoic and Mottled, 5 = Hypoechoic, 6 = Enlarged and Hypoechoic, 7 = Core Lesion, 8 = Anechoic. Mottled echogenicity refers to an irregular, patchy mix of hypoechoic and hyperechoic areas within the body of the suspensory ligament. Hypoechoic refers to a broader area that has overall reduced echogenicity compared with other areas of the ligament. A core lesion is a discreet lesion with hypoechogenicity to anechogenicity. Anechoic refers to a fluid echogenicity in a broad area of the ligament.

### 2.4. Surgical Treatment: Desmoplasty with Fasciotomy

DF was performed by two surgeons (NAW, JGB) as described by Hewes and White [21]. Briefly, horses were placed under general anesthesia in dorsal or lateral recumbency. The metatarsal regions were prepared for surgery. A 7 MHz linear ultrasound probe^1^ was placed on the medioplantar aspect of the limb to obtain a transverse view of the proximal suspensory ligament. A number 11 scalpel blade was used to make a small skin incision and a custom-made ligament splitting knife was advanced into the ligament, under ultrasonographic guidance and directed into hypoechoic and anechoic core lesions. The blade was moved in a proximal to distal direction parallel to the fibers of the suspensory ligament and the overlying plantar suspensory fascia. Additional stab incisions were made every 2 cm to make a linear split in ligament and fascia to treat all affected regions. The small incisions were left to close by second intention; sterile bandages were placed prior to recovery from general anesthesia.

Recommended postoperative care for DF included maintenance of a bandage for 4 weeks, phenylbutazone (2.2 mg/kg) orally twice daily for five days, and stall rest. Stall rest consisted of no walking for 4 weeks followed by 10 min of walking twice daily for an additional 4 weeks. Evaluation by ultrasound was recommended after 8 weeks to determine when exercise could increase. Horses were exercised at the walk under saddle for one month followed with trotting after 3 months and exercise increased thereafter. This longer rehabilitation period was due to the longitudinal lesion created in the suspensory ligament during surgery. Thus, the surgery and post-op management are a treatment regimen as a whole.

### 2.5. Surgical Treatment: Neurectomy with Fasciotomy

NF as reported by Toth et al. was performed by three surgeons (two authors MNA, JGB and a third surgeon at the hospital) as reported by Toth et al. [22]. Briefly, the horses were placed under general anesthesia and positioned in dorsal recumbency with the hindlimbs suspended. After routine preparation, a 6 cm skin incision was made lateral to the border of the superficial digital flexor tendon, centered 1.5 cm distal to the proximal aspect of the fourth metatarsal bone. The incision was continued through the flexor retinaculum and connective tissue lateral to the superficial and deep digital flexor tendons using Metzenbaum scissors. A 4 cm section of the lateral plantar nerve was identified and separated from surrounding tissue using a hemostat. A hemostat was used to isolate the DBLPN and a 2–4 cm section of the nerve was sharply excised. The proximal border of the plantar suspensory fascia was then identified and the fascia longitudinally split for 4 cm using Metzenbaum scissors. The flexor retinaculum was closed with 3-0 polyglactin 910 in a simple continuous pattern. The subcutaneous tissue was closed with 2-0 polyglactin 910 in a simple continuous pattern. The skin was closed with 2-0 nylon in a simple interrupted pattern. Sterile bandages were placed prior to recovery from general anesthesia.

For postoperative care after NF, phenylbutazone (2.2 mg/kg) was administered orally twice daily for five days. Doxycycline (5.0 mg/kg) was administered orally twice daily for five days. The limbs were bandaged and changed every 2–3 days until suture removal 14 days after surgery. Horses were confined to a stall for 30 days, followed by 30 days of small paddock turnout. A recheck examination was performed 60 days after surgery. If the horse was sound at that time, they were returned to work, with a gradual increase in exercise. The shorter rehabilitation period for NF was due to not causing a new lesion in the suspensory ligament during surgery. Thus, the NF treatment protocol as a whole is being compared against the DF treatment protocol as a whole.

### 2.6. Case Follow-Up

Medical records from the surgical center and referring veterinarians were reviewed to determine outcomes. Referring veterinarians, owners, and trainers were contacted 1–3 years after surgery. Information collected included if the horse was sound during veterinary examination, duration of time from surgery to return to unrestricted activity (full work), if the horse was able to return to higher, or intended level of use or a lesser level, the reason the horse was not able to return to the desired level of work, and complications. Pre-operative veterinarians and post-op veterinarians were the same assessors for each individual horse.

### 2.7. Statistical Analysis

To determine whether there was treatment bias, age, lameness grade, limb affected (bilateral, LH, RH), diagnostic test performed (nuclear scintigraphy, radiography, MRI), and US scores were tested using a logistic regression for treatment assignment. We used Chi-square and logistic regression to test whether treatment (NF vs. DF) predicted outcomes: sound vs. not sound; and return to work at the intended-or-higher level, at a lower level, or failed to return to work. Ultrasound severity score and bilateral versus unilateral were included as covariates, and treatment × severity and treatment × bilateral were included as interactions. To address treatment-selection bias, inverse probability weighting was calculated from baseline factors (US score, age, lameness grade, bilateral) and applied using a propensity score. Analyses were conducted using JMP Pro 18 (Statistical Discovery LLC, Cary, NC, USA) and significance set as *p* < 0.05.

## 3. Results

During the study period, 141 horses had DF (95) or NF (46) for hind limb proximal suspensory desmopathy (Table 1). Breed was identified in 140 cases and was predominately Warmblood (73) or Thoroughbred (37). Gender included 103 geldings, 35 mares, and 3 stallions. There was no significant difference between groups for the mean age (9.82 years, *p* = 0.17), gender (*p* = 0.41), or breed (*p* = 0.11). Athletic use of horses was recorded in 65 cases: 33 jumping, 18 dressage, 6 eventing, 2 fox hunting, 1 racing, 2 western pleasure, 2 trail riding, and 1 pleasure. The American Association of Equine Practitioners’ lameness grade was recorded pre-operatively in 114 horses, ranging from 0 to 4 out of 5 with a median grade of 2 for both treatment groups. Lameness duration prior to diagnosis was recorded in 50 cases and ranged from 0 to 549 days.

Ultrasonographic findings were recorded in 129 horses (Table 2 and Table 3). Horses treated with DF had significantly higher ultrasound severity scores compared to horses treated with NF (*p* = 0.001). Four horses had evidence of injury in suspensory branch(es) in addition to the proximal suspensory ligament, and all 4 of those horses were treated with DF. Logistic regression confirmed US score (*p* = 0.001), and bilateral disease (*p* = 0.0067) were significantly associated with DF. Age, duration of lameness, and lameness grade were not associated with treatment choice.

Surgery was performed bilaterally in 90 horses, the right hind only in 27 horses, and the left hindlimb only in 22 horses. Complications following DF occurred in 1 horse that had swelling. Complications following NF occurred in 3 horses and included cellulitis (1), incisional drainage (1), and heat (1) at the incision. There was no significant difference in complication rate between DF and NF.

Post-operative soundness was recorded for 109 horses, 89 horses became sound postoperatively, including 61 treated with DF (79%) and 28 treated with NF (88%). There was no significant difference in soundness between procedures (*p* = 0.30). Logistic regression adjusting for ultrasound severity confirmed no effect of treatment (*p* = 0.54). Neither lameness grade prior to surgery (*p* = 0.97), lameness duration prior to surgery (*p* = 0.32), nor the limb(s) affected (*p* = 0.18) were significantly associated with outcome. Age was significantly associated with poorer outcome (*p* = 0.013).

Long-term performance outcomes were available for 104 horses. Overall, 90 horses returned to work, 57 at the intended level and 33 at a lower level (Table 3). Of the 33 horses that returned to a lower level of work, 14 were reportedly due to suspensory ligament problems. Of the 15 horses that did not return to work, 11 were reportedly due to suspensory ligament problems.

Of the horses treated with DF, 67% returned to full work when eliminating cases with reasons other than suspensory injury. All together, 39 (53%) returned to the intended level, 22 (30%) returned at a lower level—10 due to suspensory injury, and 13 (18%) failed to return to athletic use—9 due to suspensory desmopathy. Of the horses treated with NF, 75% returned to full work when eliminating cases with reasons other than suspensory injury. All together, 18 (58%) returned to the intended level, 11 (35%) returned at a lower level—4 due to suspensory injury, and 2 (6%) failed to return to athletic use—2 due to suspensory injury.

There were no significant differences between DF and NF for returning to intended level, returning at a lower level, or failing to return due to suspensory ligament problems (*p* = 0.40). DF horses took significantly longer to return to work in univariate analysis (307 ± 154 days compared with 203 ± 101 days, *p* = 0.003), but this was not significant in the adjusted regression model (*p* = 0.29). Two horses developed suspensory breakdown (one after DF, one after two NF procedures), and one horse had increased fetlock hyperextension after DF.

Horse use was associated with outcome. Sport horses (jumping, dressage and eventing) were more likely to return to work than the other categories combined (*p* = 0.0016).

Seven horses had non-surgical treatment prior to surgery. Three horses had rest, one ECSWT, and seven a combination of treatments, most commonly rest, NSAID therapy and ECSWT. Thirty-five horses had additional postoperative treatment including intralesional injection with mesenchymal stem cells in platelet rich plasma (PRP) (2), intralesional injection with PRP (17) and ECSWT (16). Horses treated with DF were significantly more likely to have adjunctive treatment (*p* = 0.012). Neither PRP nor ECSWT were associated with differences in outcome (*p* > 0.50).

## 4. Discussion

Suspensory DF and NF have been previously described with resolution of lameness and return to the previous level of performance in 82% and 75% of horses, respectively [15,21]. Performing neurectomy of the deep branch of the lateral plantar nerve has been suggested to ameliorate pain due to nerve compression [22]. Alternatively, atrophy of muscle fibers within the proximal suspensory after neurectomy could help relieve compartment pressure [24]. The common feature of both surgical procedures is the fasciotomy, which hypothetically can relieve pressure within the compartment made up by the 2nd, 3rd, and 4th metatarsal bones and fascia on the proximal plantar surface of the ligament. Interestingly, the use of fasciotomy alone or neurectomy alone have not been reported in the literature for treatment of proximal hindlimb suspensory desmopathy, but both are currently used in our hospital, along with neurectomy with fasciotomy. We are no longer performing desmoplasty. This retrospective was performed (and data were collected) prior to switching surgical approaches in our hospital.

The results of this study showed similar return to soundness (DF 79%, NF 88%) and intended level of performance for NF (75%), but a lower return to the intended level of performance after DF (67%). Dyson and Murray reported 77.8% of horses treated with NF returned to full athletic function for at least one year [15]. Seventy percent of these horses started work two months after surgery. In our study, 75% of horses returned to full work following NF using their criteria. Thus, results for NF seem to be consistent between studies. Hewes and White reported 87% of horses with hindlimb suspensory core lesions treated with DF were able to return to full work [21]. These horses also returned to work after a mean of 7.5 months following the procedure. The lower numbers in the Hewes and White study may account for this difference. Surgery failed to resolve lameness in 20 horses overall, with 17 failures due to continued suspensory desmopathy. Adhesions of the suspensory ligament to surrounding soft tissue structures may represent a surgical complication contributing to persistent lameness [25].

Horses in the present study treated with DF returned to work approximately 10 months after surgery, which in univariate analysis was significantly longer than horses treated with NF, 7 months (*p* = 0.0023). The post-op protocol for DF recommended a minimum of 4 months of gradually increasing exercise before initiating a return to full work, based on evidence of healing of the core lesion on ultrasound, whereas the NF rehabilitation protocol was to gradually increase work after 2 months, similar to the recommendations by Dyson and Murray [15]. The longer rest period recommended after DF may partly explain the extended rehabilitation time, though it remains unclear whether this delay conferred any clinical benefit. Although not recorded, season of the year or owner decisions may also have affected when horses were placed back in full work.

Limitations of this retrospective study include incompleteness of medical records, recall bias, and lack of randomization of treatment. As a consequence, there were significant treatment biases noted, in particular the horses with worse ultrasonography scores were treated with DF, and horses treated with DF had longer rehabilitation periods. It would also have been preferable to have MRI as the diagnostic imaging method of choice; however, only one horse had this technique performed in this population. Inclusion of adjunct treatments such as platelet rich plasma injection may also have affected results.

Dyson and Murray [15] identified excessively straight hock conformation and/or hyperextension of the metatarsophalangeal joints as a risk for lack of resolution of hindlimb proximal suspensory desmopathy after surgery. Horses with this conformation, as well as hindlimb proximal suspensory desmopathy, were not able to return to work compared to 77.8% of horses without this conformation [15]. Concurrent sources of lameness also had a negative effect on the outcome. Horses in our study had no concurrent lameness reported at the time of surgery, and hindlimb conformation was not always noted in the medical records. It is possible that some horses with straight tarsi and/or hyperextended metatarsophalangeal joints were treated surgically, thereby contributing to fewer horses returning to full use.

Two important differences existed between the two surgical groups. Firstly, horses treated with DF had significantly higher ultrasound severity scores compared to those treated with NF, indicating that DF was preferentially selected for more severe cases (Table 2) (*p* = 0.001). This aligns with the original description of DF by Hewes and White, who proposed the technique for horses with core lesions as a method to decompress acute lesions and enhance blood supply to chronic injuries [21]. In contrast, NF has been described to decompress the suspensory ligament [22] and cause neurogenic atrophy of intraligamentous muscle fibers [24]. Despite the bias toward using DF in horses with more severe ultrasonographic abnormalities, treatment type was not associated with differences in postoperative soundness or return-to-work outcomes. Ultrasound severity itself was also not significantly predictive of outcome, reinforcing that once severity is accounted for, surgical technique does not appear to influence prognosis.

Horses treated with DF were more likely to receive adjunctive non-surgical treatments such as platelet-rich plasma, stem cells, or extracorporeal shockwave therapy. While these were presumably targeted toward the more severely affected individuals, their use did not translate into improved outcomes in the present cohort. The only factor significantly associated with outcome was age, with older horses more likely to fail to return to work due to suspensory disease.

## 5. Conclusions

In conclusion, this study indicates that there were no significant differences between NF and DF as treatments for hindlimb suspensory desmopathy in terms of resolution of lameness or return to work. Although DF was preferentially used in cases with more severe ultrasonographic findings, outcomes were similar between groups. Horses treated with DF were treated with significantly longer rehabilitation, while NF was associated with faster return to work but may carry risk of incisional complications. DF remains effective for treating core lesions, though its broader benefit relative to NF remains uncertain.

## Figures and Tables

**Table 1:** Summary of pre-operative and outcome data.

	Number	Age (Years)	Gender	Limb Affected	Lameness	Lameness Duration (Days)	Sound Post-Op	Time Until in Work (Days)	Return to Work (All Cases)
ALL	141	9.8 (3.4)	103 Geldings 35 Mares 3 Stallions	90 Bilateral 23 Left 28 Right	2 [0–4]	146 (182)	71 (71%)	273 (147)	90 In Work (86%) 57 Intended Level 33 Lower Level
DF	95	9.6 (3.5)	68 Geldings 24 Mares 3 Stallions	68 Bilateral 13 Left 14 Right	2 [0–4]	162 (155)	48 (69%)	307 (154) *	61 In Work (82%) 39 Intended Level 22 Lower Level
NF	46	10.3 (3.6)	35 Geldings 11 Mares 0 Stallions	22 Bilateral 15 Left 9 Right	2 [1–4]	127 (208)	23 (77%)	203 (102) *	29 In Work (94%) 18 Intended Level 11 Lower Level

Number (%), average (standard deviation) median [range] are presented. Asterisk denotes significant differences.

**Table 2:** Summary of ultrasonographic grading and treatment choice.

Treatment	Mean US Score	SD	Median	Range	*p*-Value NF vs. DF
All horses	4.8	2.1	5	0–8	—
DF	5.4	1.9	6	1–8	0.001
NF	3.2	2.1	3	0–6	0.001

Mean ultrasonographic score (standard deviation), median ultrasonographic score (range) and *p*-values are listed above. There was a significant difference between groups for US score.

**Table 3:** Ultrasonographic score, treatments, and outcomes for return to work.

Outcome	DF (Mean ± SD)	NF (Mean ± SD)	*p*-Value
**All Horses**	5.4 ± 1.9	3.2 ± 2.1	0.54
**Return to intended level**	5.4 ± 2.0	3.6 ± 2.2	0.42
**Return at lower level**	5.7 ± 1.5	3.2 ± 2.1	0.48
**Failed to return (suspensory)**	5.6 ± 1.9	4.3 ± 1.8	0.57

Performance outcomes are listed, and compared between DF and NF, with *p*-values for return-to-work associations shown in the column on the right. No significant differences were found between treatment types, and ultrasonography scores with respect to return-to-work.

## Data Availability

The original contributions presented in this study are included in the article. Further inquiries can be directed to the corresponding author.
